# Dentition and Its Derangements. Lecture X, Part 1

**Published:** 1862-10

**Authors:** A. Jacobi


### Selections.

---

Dentition and its Derangements—*By A. Jacobi*, M. D. —*LECTURE X*—Part 1.—About or a little after their first year, children begin to walk; such as are backward will be excused by the mother with their teething. Others commence to walk at, or soon after, the regular time, but their lower extremities, perhaps also the forearms and other bones, will undergo very severe curvatures. Others walk at the right time, have their teeth when they ought to, but the sutures of the cranium do not close in time, the occipital portion of the cranium is softened, and symptoms of exudative meningitis show themselves. This is popularly believed to be from teething. Others have a somewhat compressed thorax, the sternum being prominent, the ribs flattened, and the points of insertion of the ribs at the sternum greatly swollen. This form of thorax, commonly called "chicken breast," is also said to owe its origin to "teething." And there will be a great many cases in adults, of sooliotic or kyphotic curvatures of the vertebral column, which will, either by themselves or others, be attributed to difficult teething in infancy.

The series of symptoms and affections which I have hitherto mentioned have certainly a common cause, but whether teething is this cause, is another question. They, together with a number of other symptoms, constitute what has been named rhachitis, or rickets, the most constant and remarkable anatomical symptoms of which are the following:

1. Enlargement of the epiphyses of the long tubular bones.
2. Softening of the osseous tissue generally.
3. Thickening of flat bones, skull, scapula, etc.
4. Deformities resulting from mechanical causes influencing the softened bones of the chest, pelvis, vertebral column, and the tubular bones. These mechanical causes are principally the action of the muscles, and the weight of the body.
5. Retardation of growth, in both bones and such portions as are in anatomical or physiological connection with the bones: for instance, muscles, blood-vessels, nerves and teeth.
6. Certain alterations in the pericardium, lungs and integuments of the spleen, partly to be explained by the pressure of the deformed thorax.

7. Less constant but highly important alterations in the nutrition of brain, spleen, liver, lymphatic glands, muscles, and other organs.

Rhachitis is eminently a disease of the infantile age, and is very rarely—in truth only in a few cases, part of which are even doubtful—found immediately after birth. The first symptoms show themselves at the age of a few months or years. A few statistical statements will suffice to give at least some information concerning the connection between rhachitis and, particularly beginning, dentition.

Guérin found, in 346 cases of rhachitis, right after birth, 3; under one year, 98; under two years, 176; under three years, 35; under four years, 19; under five years, 10; under twelve years, 5, of this affection.

Luszinsky has the following percentages:—Under six months, 13; in another year, 20; under twelve months, 25; from one to three years, 56; from four to six years, 5; from seven years upwards, 1.

Of 1,654 rhachitical patients observed by Küttner, there were

| | Males. | Females. | Total. |
|---|---|---|---|
| Under 6 months, | 14 | 4 | 18 |
| From 6 to 9 months, | 31 | 18 | 49 |
| " 9 months to 1 year, | 67 | 53 | 120 |
| Under 1½ years, | 193 | 165 | 358 |
| " 2 " | 151 | 186 | 337 |
| " 3 " | 215 | 234 | 449 |
| " 4 " | 81 | 97 | 178 |
| " 5 " | 28 | 44 | 72 |
| " 6 " | 13 | 11 | 24 |
| Over 6 " | 21 | 28 | 49 |

The largest proportion of cases occur in the second year, after the protrusion of teeth has fairly commenced. The first weeks of life are almost exempt, but there have been described cases of not only early but even fœtal development of rhachitis. Under eighteen months, the male sex appears to have the larger number of patients, while towards the close of the second year and later, the female appears to be more affected.

Extensive chemical investigations have led to the following results: that the general chemical character of rhachitical bones consists in too small an amount of the earthy salts. It is least during the height of the process, the remaining portions of the old tissue being more similar to the normal condition; the new formed "osteoid" parts, however, showing the greatest deviations. In these the amount of carbonic acid is also increased. The percentage of fat shows very little difference; indeed, with the exception of the long tubular bones, in which a large amount of fat is found in consequence of the soft medullary substance, both in the spongiose part of the bones and the dilated medullary canal. The specific gravity of rhachitical bones is generally diminished in proportion to the intensity of the process. The organic basis of the tissue has undergone no essential alteration, but the non-ossified cartilages have increased their proportion of water. The old question, whether the rhachitical bone yielded chondrin like cartilage, or gluten like bone, has finally been answered by the discrepancies of opinions being explained by the different and partially defective modes of inquiry. There is gluten, not chondrin, in rhachitical bone, which therefore ranks, not among cartilaginous tissue, but at the side of normal bone; while at the same time the increased amount of carbonic acid found in the recent rhachitical deposits places them side by side with the normal physiological process of growth.

As principal causes of rhachitis, many authors have considered either diminished introduction, or impaired assimilation, or increased elimination of phosphates. That the former assumption can not be sustained is proved by the large amount of phosphates contained in every one of the articles of food in common use. Impaired assimilation depends on a diseased condition of one or more of the digestive organs, which, it is true, are always found disordered in a certain stage and development of rhachitis. Increased elimination of phosphates through the urine, according to Kletzinsky through the fæces also, is generally found to take place, but not always. Exceptions have expressly been stated—for instance, by Dr. Friedleben, who has found a number of instances of well marked rhachitis with no increase of the amount of phosphates in the renal secretion. He, moreover, points to the fact, that the presence of a surplus of phosphates in the urine, when proved, may just as well be the consequence as the cause of the rhachitical process. For, certainly, where ossification is prevented from taking place normally, the phosphates have to be removed somewhere from the blood containing too large an amount of them.

Altered condition of food, particularly of the proteinates, and disorder of one or more of the important digestive organs, at a period of life in which the organism requires much and appropriate new material, are the prominent first causes of rhachitis. At the same time all such diseases and disorders which may lead to impaired assimilation and nutrition will help in bringing on rhachitis. Thus Dr. Friedleben lays particular stress on the importance of early diseases of the respiratory organs. It is true, that almost all the post-mortem examinations of rhachitical children have yielded collapse, induration, or atelectasis of the pulmonary tissue. These alterations must not be explained by the pressure of the abnormally curved ribs and costal cartilages, for these alterations are frequently found where there can be no pressure from the ribs; and experience teaches, that even the most acute processes, like pneumonia, will result in depressions and irregular configurations of the thorax. Thus in many cases not the rhachitical process of the ribs, but the morbid condition of the lungs, must be taken as the original source of asymmetry, depression, and narrowness of the chest. The frequency of pulmonary or bronchial affections, before or during the rhachitical process, appears to justify the assumption of a direct connection between them; any serious either acute or protracted affection of the respiratory organs, necessarily involving an impaired condition of the blood from which every new tissue is to be formed. Just add thereto insufficient food wanting in proteine, narrow room, overcrowded population, bad air, and careless nursing, and you will no longer wonder at the combined result showing itself in the abnormal growth of the infantile cartilage and bone.—*American Medical Times.*

